# Pasting and Gel Behavior of Durum Wheat Derivatives

**DOI:** 10.3390/gels11120991

**Published:** 2025-12-10

**Authors:** Diogo Salvati, Laura Moreno, Juan Manuel Antolín-Rodríguez, Manuel Gómez

**Affiliations:** Food Technology Area, College of Agricultural Engineering, University of Valladolid, Av. Madrid, 34004 Palencia, Spain; diogo.salvati@uva.es (D.S.);

**Keywords:** RVA, pasting, texture, durum wheat, by-product

## Abstract

Durum wheat (*Triticum durum*) is one of the main raw materials in the food industry, used primarily in the production of pasta. During milling, semolina and flour are obtained with different size distributions, and different compositional and functional characteristics, which influence processes such as gelatinization, retrogradation and the final texture of the products. Understanding these changes is essential for optimizing the technological quality and shelf life of processed foods. The aim was to evaluate how particle size, composition, temperature, and treatment time affect gelatinization, retrogradation, and gel texture. Samples included common wheat flour (control), durum wheat semolina, durum wheat flour, and re-milled semolina (<180 μm). Hydrothermal tests were conducted at 95 °C with varying holding times, and at 140 °C with extended cooling to observe retrogradation. Composition and particle size were found to determine rheological behavior. Semolina showed higher retrogradation and produced firmer gels, while durum wheat flour, with higher protein and ash content, showed atypical profiles and less consistent gels. Increased temperature and time enhanced breakdown and reduced final viscosity, indicating starch thermal degradation. A correlation was observed between final viscosity and gel hardness. This study provides information useful for optimizing the milling, cooking, and development of durum wheat-based products with improved texture and shelf life.

## 1. Introduction

Starch is the main component of cereals and their derivatives, such as flours and semolinas. This component, composed of amylose and amylopectin, is capable of gelation upon hydration and heating, followed by retrogradation upon cooling, forming gels [1]. The behavior of starch-rich products during this phase depends on the amylose and amylopectin content and fine structure of these components [2], the proportion of damaged starch and particle size [3], as well as other constituents [4]. This behavior is crucial for predicting the organoleptic characteristics of various food products, as well as their glycemic index [5,6].

Durum wheat (*Triticum durum*) is the raw material for the production of wheat semolinas, which are the main ingredient in pasta manufacturing [7]. Semolina milling industries generate smaller fractions of flour, which have a finer particle size than semolinas [8]. The inclusion of these flours in pasta production results in lower-quality products [9], and thus they are typically separated and used as a by-product for animal feed [8]. Due to the semolina production process, which differs from that of flours, these flours do not undergo treatment to remove bran residues, and their proximate composition differs from that of semolinas, which influences their pasting properties. It is also possible to re-mill semolinas to produce flours intended for bread making, a practice common in Italy, where durum wheat consumption is higher [10,11]. However, these flours contain a high proportion of damaged starch and higher protein content, which affects their pasting properties compared to flours obtained from soft wheat.

To study the pasting properties of starch-rich products, instruments such as viscometers with temperature ramping capabilities, including the Rapid Visco Analyser (RVA), are commonly used. Analyses are typically conducted following official methods [12]. In these methods, the sample is hydrated with a specific amount of water, heated to 95 °C, held at that temperature for a defined period (2.5 min) to complete gelatinization, and then cooled to 50 °C, maintained for 2 min to analyze retrogradation. However, processes such as extrusion expose samples to higher temperatures and pressures, and it has been demonstrated that the pasting behavior of starch-rich products under these conditions changes [13,14]. Heating time is also known to influence thermal starch degradation, which can affect subsequent processes such as retrogradation, although this aspect has not been fully studied.

Our hypothesis is that both the type of product obtained from durum wheat (semolina, flour, or re-milled semolina) and the heating and cooling process will significantly affect pasting properties and the characteristics of the resulting gels. To test this, three products derived from durum wheat (semolina, flour, and re-milled semolina) will be compared with a soft wheat flour. All products will undergo a standard heating and cooling cycle, as well as additional cycles with varying heating durations and one reaching 140 °C. Viscosity will be analyzed during these cycles, and the texture of the resulting gels will be evaluated.

The novelty of this work is threefold. First, it represents the first study of the pasting properties of durum wheat flours. Second, it is the first time that the effect of treatment time on pasting properties has been investigated. Finally, this is the first study to examine how pasta behavior under prolonged cooling correlates with the texture of the resulting gels.

## 2. Results and Discussion

**Figure 1:**
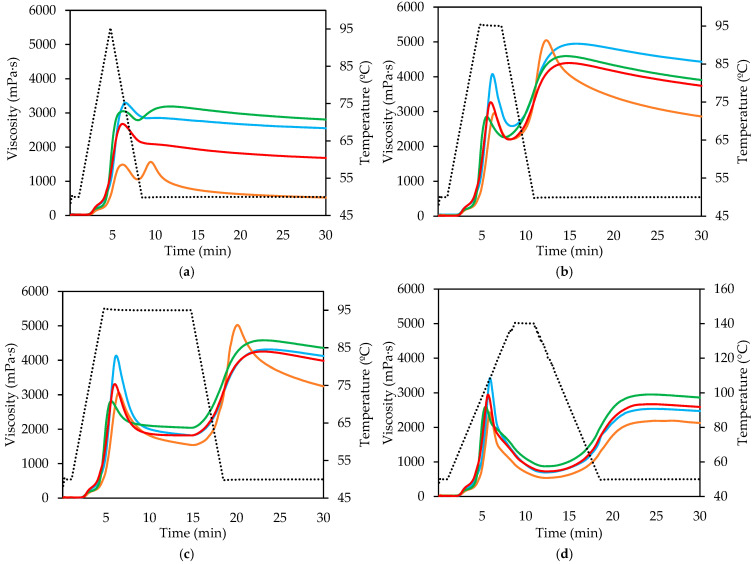
Viscosity profile of flours and semolina: (**a**) minimum treatment time (0.01 min at 95 °C); (**b**) standard treatment (2.50 min at 95 °C); (**c**) maximum treatment time (10.03 min at 95 °C); (**d**) high-temperature treatment (2.50 min at 140 °C). Temperature profile (dashed line); Common wheat flour (blue curve); Durum wheat flour (orange curve); Durum wheat semolina (green curve), and Re-milled durum wheat semolina (red curve).

In Figure 1, distinct pasting profiles were observed among the different flours and semolina samples (see Supplementary Materials for details), highlighting the influence of flour type and thermal treatment on starch gelatinization and subsequent viscosity development. In both the control and over-treated curves, common wheat flour (CWF) exhibited the highest peak viscosity. This behavior is attributed to its lower protein content and consequently higher starch content, which promotes granule swelling and amylose leaching during heating, thereby enhancing paste viscosity [15].

Among the durum wheat samples, the re-milled semolina (SRM) produced a slightly higher peak than the standard durum wheat flour (DWF). This is probably due to the low protein and therefore high starch content in SRM, since starch is the component that retrogrades and is responsible for the peak. The higher ash and protein content of DWF indicates the presence of bran residues, which slightly reduces the starch fraction but increases the heterogeneity of the matrix, influencing water distribution during heating. In contrast, the durum wheat semolina (DWS) displayed the lowest peak viscosity values, indicating restricted water diffusion into the dense and compact particles typical of semolina. The reduced porosity and higher packing density of these particles limit starch granule expansion and swelling, resulting in a lower viscosity increase during heating [16,17].

Lower treatment intensity resulted in a lower viscosity peak, indicating that complete starch gelatinization does not occur. This effect is more pronounced in DWF, which coincides with the fact that the peak observed in the standard curve for this sample was reached later than in the other samples. This indicates that bran residues and their components impede and delay the gelatinization process. The higher proportion of non-starch components in DWF (bran contamination) competes with starch granules for available water, thereby reducing starch hydration, delaying gelatinization onset, and limiting granule swelling. As a consequence, the paste achieves a markedly lower peak viscosity [18]. This competitive water binding and the steric hindrance imposed by non-starch macromolecules have been previously identified as major factors that decrease paste viscosity and modify starch gelatinization kinetics [16,19].

Regarding the breakdown parameter, it is higher in CWF than in SRM, especially when treatment time or temperature increases, in which cases the viscosities of both tend to converge. In DWF, the behavior is similar, showing a higher breakdown when the heating time is increased to 95 °C and a considerable decrease, which is not recovered with high-temperature treatment. Conversely, DWS showed the lowest breakdown values, indicative of high thermal and mechanical stability. The compact particle morphology and reduced internal porosity restrict water penetration, preventing plasticization of amorphous regions and maintaining granule integrity throughout heating [16]. Consequently, viscosity loss during the holding phase is minimized. These results demonstrate that granule breakdown is strongly governed by matrix architecture and water mobility rather than starch composition alone. Under the shorter thermal treatments, minimal breakdown was observed across all samples, as the viscosity peak was reached near the end of the heating cycle, leaving little time for granule disruption to occur.

Regarding retrogradation, both the standard and extended treatment samples of CWF and SRM exhibited similar increases in viscosity during cooling, indicating comparable tendencies toward re-association of starch molecules. However, in the standard treatment, CWF attained a higher final viscosity because it began the cooling phase at a higher viscosity level. Under prolonged treatment, these differences diminished as the prior viscosities converged. In contrast, DWS showed a more pronounced setback, suggesting that the limited granule degradation during heating preserved a greater potential for reassociation upon cooling [20]. This behavior indicates that the granules retained sufficient internal order to facilitate amylose and amylopectin recrystallization during the cooling phase.

DWF exhibited a particularly distinctive profile, characterized by a rapid rise to the initial peak followed by a sharp viscosity decline. Extending the cooling period revealed a subsequent partial recovery, consistent with the formation of amylose–lipid complexes within the matrix [21]. A similar post-peak viscosity decrease was observed, albeit to a lesser extent, in the CWF, DWS and SRM samples.

At high temperature and pressure (2.50 min at 140 °C) (Figure 1d), where starch granules were extensively degraded, no notable differences in setback among the products were detected. In this case, DWS presented the highest final viscosity, consistent with its lower breakdown and greater structural stability. For the mildly treated samples, either no retrogradation (CWF, SRM) or minimal retrogradation (DWF, DWS) was detected, indicating that incomplete gelatinization and granule disruption prevent the reassociation of starch chains during cooling. The marked viscosity reduction observed for DWF relative to the other flours underscores the role of native protein and cell-wall matrices in stabilizing the paste. These structures likely form elastic networks that absorb mechanical stress and prevent rupture of swollen granules during heating and shearing, as previously described for cereal systems [19].

**Figure 2:**
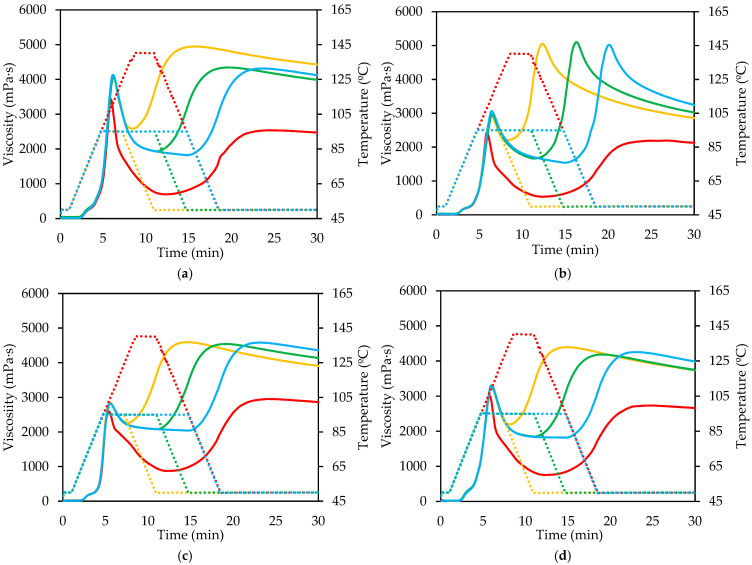
Viscosity profile of different flours and semolina treated at high temperature and at 95 °C with extended treatment time: (**a**) Common wheat flour; (**b**) Durum wheat flour; (**c**) Durum wheat semolina, and (**d**) Re-milled durum wheat semolina. Red: high temperature (140 °C); Yellow: 2.50 min; Green: 6.96 min; Blue: 10.00 min. Temperature profile (dashed line).

**Figure 3:**
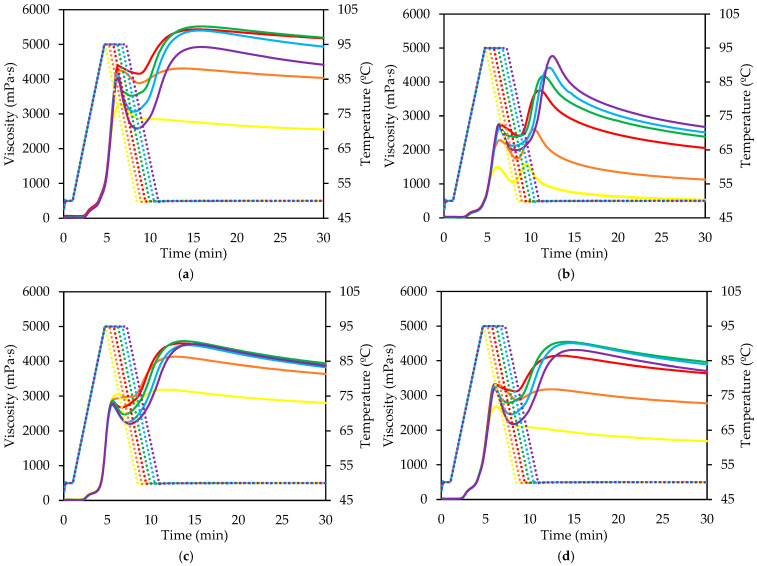
Viscosity profile of different flours and semolina treated at 95 °C with reduced treatment time: (**a**) Common wheat flour; (**b**) Durum wheat flour; (**c**) Durum wheat semolina, and (**d**) Re-milled durum wheat semolina. Yellow: 0.01 min; Orange: 0.50 min; Red: 1.00; Green: 1.50 min; Blue: 2.00 min; Violet: 2.50. Temperature profile (dashed line).

Figure 2 and Figure 3 show pasting curves for the same product under extended (Figure 2) or reduced (Figure 1) thermal treatment. It is evident that longer treatment increases both breakdown and retrogradation, indicating a correlation between the two phenomena. In DWS and SRM, final viscosities tend to converge, except for high temperatures (140 °C), compensating for the higher breakdown in prolonged treatments. In CWF, a similar trend is observed, but final viscosities do not fully converge; higher breakdown correlates with lower final viscosity. This indicates that, under atmospheric pressure, greater rupture and loss of integrity of the starch granules facilitates the reorganization of the fractions released during the process, which in turn generates a higher viscosity after cooling.

DWF showed similar behavior, with a high initial retrograde peak, similar in all cases with heating times higher than 2.5 min (Figure 2b). Conversely, under shorter heating times (Figure 3b), exhibited an opposite, lower breakdown values were associated with more pronounced setback and higher final viscosities. This indicates that moderate granule integrity favors partial amylose diffusion while maintaining sufficient structural order to promote effective chain reassociation upon cooling [22].

Curves under high temperature and pressure show lower peaks because starch granule degradation begins during heating, causing some breakdown, which is more pronounced during the holding phase compared to curves reaching only 95 °C. Retrogradation is similar across all products and comparable to curves under milder heating, but final viscosity is lower due to reduced post-heating viscosity. All samples showed reduced peak viscosities compared with those treated at 95 °C. This decrease reflects early starch degradation and partial dextrinization occurring during the heating stage, which reduces granule swelling capacity and weakens paste viscosity [23]. The breakdown was more pronounced during the holding phase, resulting from a thixotropic behavior that facilitates material flow and reduces viscosity over time [24], alongside concurrent starch degradation [23]. Although retrogradation magnitudes were comparable among the samples and similar to those observed under milder conditions, the final viscosities were considerably lower. This reduction is associated with the limited ability of fragmented starch chains to reorganize into crystalline regions during cooling [16,25]. The high degree of depolymerization and disruption under these extreme conditions prevents the formation of a continuous network, resulting in weak gel structures upon cooling.

Under minimal heating, clear differences are observed, particularly in the shortest treatments, where limited starch degradation (low breakdown) results in negligible retrogradation and minimal final viscosity increase during cooling.

Regarding gel hardness (Table 1), a significant correlation (r = 0.7, *p* < 0.001) with final viscosity was observed. Notably, gels made from DWF exhibited the lowest hardness and reduced final viscosity values. But the hardness of these gels was also lower than that of those obtained with other products and treatments showing the same or even lower final viscosity. This indicates that bran residues interfere with gel network formation, weakening the structure. In the case of shorter heating times and less starch gelatinization, it was not possible to obtain gel hardness. Despite demonstrating notable final viscosity levels, the samples exhibited an inability to form gels capable of sustaining themselves and preventing compression, thereby hindering the discernment of a hardness peak. The absence of consistency in the samples was attributed to constrained retrogradation (less setback) and reduced final viscosity values. In the DWS, the firmest and most opaque gels were obtained after cooling, in accordance with a more pronounced retrogradation, which could be related to their coarse particle size and greater reorganization of the amylose chains [26].

Extending the RVA cooling time reveals this weakening, which is not evident in the standard test (~11 min cooling). Gels subjected to 140 °C treatment, which results in higher starch degradation and lower final viscosity values, also exhibit lower hardness, confirming the critical role of starch retrogradation in gel strength. This behavior is compatible with thermal degradation and incomplete retrogradation, as described in cooked or extruded cereals [14,27]. These results show that not only particle composition and size affect thermo-rheological properties, but also that temperature is a determining factor. High temperature conditions generate greater starch degradation, preventing the formation of stable gel structures.

## 3. Conclusions

Flour/semolina type, composition, particle size, structure, and thermal treatment collectively determine starch gelatinization, paste viscosity, breakdown, retrogradation, and gel texture. Common wheat flour (CWF) exhibited the highest peak viscosity due to its lower protein content and higher starch availability. In contrast, durum wheat flour (DWF), with bran particles, displayed a bimodal pasting profile, lower viscosity, and enhanced thermal stability, reflecting complex interactions within the starch matrix. Durum wheat semolina (DWS) showed minimal breakdown and high paste stability, attributed to its dense particle structure and restricted water penetration, while re-milled semolina (SRM) exhibited intermediate behavior influenced by particle size and matrix heterogeneity.

Extended or high-temperature treatments promoted starch degradation, reduced final viscosity, and limited retrogradation, whereas milder treatments preserved granule integrity, facilitating amylose reassociation during cooling. These findings provide valuable insights into flour and semolina functionality for high-temperature and high-shear processing, where controlling viscosity, breakdown, and retrogradation is critical for product quality.

The gels showed a correlation between final viscosity and durability; however, a longer cooling time is necessary, especially for samples with longer heating times, in order to better verify the retrogradation profile of these samples.

## 4. Materials and Methods

### 4.1. Materials

In the present study, samples of durum wheat flour (2.23% ash; 17.49% protein; 111 µm D[4,3]) and durum wheat semolina (0.91% ash; 14.69% protein; 349 µm D[4,3]) were used, both obtained from the same batch (Gallo, Barcelona, Spain).

In addition, re-milled durum wheat semolina (0.99% ash; 14.80% protein; 144 µm D[4,3]) and common wheat flour (*Triticum aestivum*) (Harinera Castellana, Medina del Campo, Spain) (0.64% ash; 11.86% protein; 141 µm D[4,3]) was used as a control sample to allow a systematic comparison with the durum flours and semolina samples.

Re-milled semolina was obtained from the original durum wheat semolina, which was further ground using a Retsch ZM 200 mill (Retsch Inc., Haan, Germany) equipped with a 200 µm sieve. The finer particles were then fractionated using a Bühler MLI300 B automatic sifter (Bühler, Braunschweig, Germany) fitted with a 180 µm sieve, in order to obtain a flour with a particle size distribution similar to that of the durum wheat flour (<180 µm).

All processed samples were stored in polypropylene bags and kept at room temperature, under dark conditions, until further analysis.

### 4.2. Methods

#### 4.2.1. Semolina and Flour Characterization

The particle size distribution of the flour and semolina samples was determined using a laser diffraction analyzer (Mastersizer 3000, Malvern Instruments, Malvern, UK). From the particle size distribution curves, the D[4,3] values were calculated.

Ash (AOAC Method 935.42), and protein (AOAC Method 945.18B) contents were determined following the official methods of the Association of Official Agricultural Chemists [12]. A nitrogen-to-protein conversion factor of 6.25 was used. The results obtained are expressed in grams per 100 g of sample on a dry-weight basis, and the experiments were performed in triplicate.

#### 4.2.2. Hydrothermal Treatment

The samples under study were hydrothermally processed using a Rapid Visco Analyser (RVA 4800, Perten Instruments Australia Pty Ltd., Sydney, Australia). For each sample, a suspension was prepared in an aluminum canister by adding 25.0 ± 0.1 g of distilled water and 4.0 ± 0.1 g of flour or semolina, adjusted to a moisture content of 14% according to the specific moisture content of each material. The standard treatment, designated curve t = 2.5, involved a holding time of 2.5 min at 95 °C with temperature cycles programmed in accordance with the general pasting method AACC n° 61-02.01 [12].

**Figure 4:**
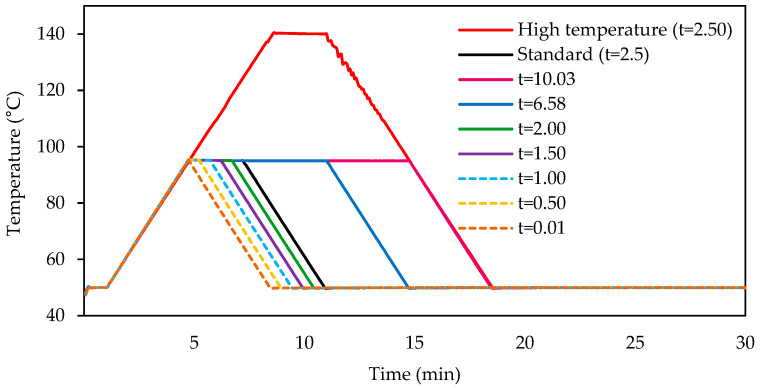
Temperature profiles applied during RVA analysis of durum wheat flours and semolina under different hydrothermal treatments.

The temperature ramp established during the experimental phase (Figure 4) consisted of an initial holding step at 50 °C for one minute, followed by heating at a rate of 6 °C per minute until the target temperature was reached. This target temperature was either 95 °C for the standard method or 140 °C for the high-temperature method. Once the target temperature was reached, the samples were held at that temperature for a predetermined duration. For the standard method (95 °C), various holding times were applied: 0.01, 0.5, 1.0, 1.5, 2.0, 2.5, 6.96 and 10.0 min. These corresponded to curves t = 0.01, t = 0.5, t = 1.0, t = 1.5, t = 2.0, t = 2.5, t = 6.96 and t = 10.0, respectively. The high-temperature profile involved holding the samples at 140 °C for 2.5 min. After this, the samples were cooled at a rate of 6 °C per minute until they reached 50 °C and were then maintained at this temperature until the total analysis time reached 30 min. All measurements were carried out in triplicate.

#### 4.2.3. Textural Properties of Gels

In order to evaluate the texture of the gels, the samples obtained from the hydrothermal treatments were deposited in circular plastic containers measuring 34 mm in diameter and 15 mm in height. The containers were then sealed with polyethylene adhesive film to prevent moisture loss. The samples were stored at 4 °C for 24 h to ensure adequate consistency and stability of the gels.

Following 24 h, the gels were extracted from the containers and subsequently subjected to a hardness test. For this study, a TA-XT2 texturometer (Stable Micro Systems Ltd., Surrey, UK) was utilized, equipped with Exponent Lite version 6 software for Windows. A test protocol was implemented that comprised a compression test, using a 75 mm-diameter cylindrical probe (P75), with a compression of 5 mm, a speed of 1 mm·s^−1^. Hardness (g) was calculated as the maximum force recorded during the compression, representing the point at which the gel exhibits the greatest resistance to deformation. The analysis was performed in duplicate for the high-temperature, standard curve, t = 10.00, t = 0.01, t = 0.50 and t = 1.00 treatments.

#### 4.2.4. Statistical Analysis

The data obtained from the different flours and semolinas were analyzed by analysis of variance (ANOVA). The statistical analysis was performed using Statgraphics Centurion XVI (Version 19.6.04) software (Statpoint Technologies, Warrenton, VA, USA). To compare means, Fisher’s Least Significant Difference (LSD) test was applied at a 95% confidence level. Pearson’s correlation coefficient (r) was calculated to assess linear associations between variables, using a significance level of 0.05.

## Figures and Tables

**Table 1:** Hardness and final viscosity parameters of durum wheat flour and semolina gels.

Sample	Treatment	Hardness (g)	Final Viscosity (mPa·s)
CWF	2.50 (HT)	328.12 ± 16.87 ^ef^	4448.50 ± 106.77 ^k^
	10.00	352.53 ± 33.77 ^fg^	4266.50 ± 198.70 ^k^
	2.50 (S)	383.16 ± 11.99 ^fgh^	4365.00 ± 11.31 ^k^
	1.00	474.05 ± 37.28 ^ij^	5167.50 ± 91.22 ^l^
	0.50	530.51 ± 53.82 ^kl^	4028.50 ± 10.61 ^j^
	0.01	291.40 ± 16.32 ^de^	2549.50 ± 112.43 ^e^
DWF	2.50 (HT)	156.20 ± 64.52 ^c^	2072.00 ± 32.53 ^d^
	10.00	64.91 ± 2.22 ^b^	3113.00 ± 206.48 ^h^
	2.50 (S)	140.05 ± 7.73 ^c^	2697.50 ± 44.55 ^ef^
	1.00	-	2043.00 ± 101.82 ^d^
	0.50	-	1155.50 ± 62.93 ^b^
	0.01	559.24 ± 7.86 ^kl^	2921.50 ± 24.75 ^g^
DWS	2.50 (HT)	368.22 ± 11.80 ^fg^	2815.00 ± 77.78 ^fg^
	10.00	473.29 ± 34.29 ^ij^	4376.00 ± 45.25 ^k^
	2.50 (S)	371.01 ± 7.67 ^fg^	3941.50 ± 44.55 ^j^
	1.00	435.39 ± 39.62 ^hi^	3986.50 ± 167.58 ^j^
	0.50	515.13 ± 2.18 ^jk^	3654.00 ± 15.56 ^i^
	0.01	559.24 ± 7.86 ^kl^	2921.50 ± 24.75 ^g^
RMS	2.50 (HT)	284.44 ± 50.68 ^de^	2607.00 ± 66.47 ^e^
	10.00	265.31 ± 20.38 ^d^	3966.00 ± 45.25 ^j^
	2.50 (S)	391.86 ± 6.46 ^gh^	3677.00 ± 114.55 ^i^
	1.00	581.41 ± 25.64 ^l^	3727.00 ± 94.75 ^i^
	0.50	473.25 ± 9.57 ^ij^	2871.00 ± 45.25 ^fg^
	0.01	252.71 ± 5.60 ^d^	1744.50 ± 4.95 ^c^

Values are expressed as mean ± standard deviation. Different letters within a column indicate significant differences (*p* < 0.05).—no detectable results; CWF: control wheat flour; DWF: durum wheat flour; DWS: semolina durum wheat; RMS: Re-milled semolina; HT: high temperature treatment; S: standard treatment.

## Data Availability

The data presented in this study are available on request from the corresponding author due to privacy.

## Supplementary Materials

The following supporting information can be downloaded at: https://www.mdpi.com/article/10.3390/gels11120991/s1, Table S1: Viscosity parameters of durum wheat flour and semolina gels.
