# Supplementary material for: Pasting and Gel Behavior of Durum Wheat Derivatives
Source: Gels. 2025 Dec 10;11(12):991. doi: 10.3390/gels11120991 (PMC12733329; doi:10.3390/gels11120991)
Supplement: Supplementary file 1 [file gels-11-00991-s001.zip › gels-3998987-supplementary.pdf]

Table S1. Viscosity parameters of durum wheat flour and semolina gels.

Sample	Treatment	Peak viscosity (mPa·s)	Breakdown (mPa·s)	Final viscosity (mPa·s)	Peak Time (min)	Pasting Temperature (°C)
CWF	**2.50 (HT)**	3980±645^{dc}	3210±581^{a}	4448±107^{c}	5.90±0.04^{ijk}	67.8±1.7^{de}
	**10.00**	4353±244^{ab}	2347±115^{b}	4267±199^{cd}	6.13±0.00^{fg}	67.7±0.0^{de}
	**6.58**	4083±120^{abcd}	2148±71^{bc}	3975±81^{efg}	6.17±0.05^{ef}	68.5±0.0^{de}
	**2.50 (S)**	4053±67^{bcd}	1509±71^{ef}	4365±11^{c}	6,13±0.00^{fg}	67,7±0.0^{de}
	**2.00**	4127±106^{abcd}	1074±115^{i}	4895±27^{b}	6.20±0.00^{def}	68,5±0.0^{de}
	**1.50**	4014±63^{cd}	548±96^{mnop}	5160±134^{a}	6,17±0.005^{ef}	68,5±0.0^{de}
	**1.00**	4383±159^{a}	226±37^{q}	5168±91^{a}	6,33±0.00^{bcd}	68,1±0.6^{de}
	**0.50**	4253±62^{abc}	370±55^{opq}	4029±11^{ef}	6.44±0.005^{bc}	68.1±0.6^{de}
	**0.01**	3302±165^{efg}	472±44^{nopq}	2550±112^{pq}	6.47±0.09^{b}	68.1±0.6^{de}
DWF	**2.50 (HT)**	2487±190no	1961±148^{cd}	2106±120^{r}	5.83±0.14^{jk}	78.3±11.9^{de}
	**10.00**	2966±249^{ijkl}	1371±96^{fg}	3113±206^{k}	6.34±0.09^{bcd}	70.5±0.6^{cde}
	**6.58**	3017±82^{ghijk}	1330±49^{fgh}	3056±71^{kl}	6.40±0.10^{bc}	70.9±1.1^{cde}
	**2.50 (S)**	2832±58^{klm}	778±80^{jklm}	2698±45^{op}	6.30±0.04^{cde}	79.5±12.1^{abc}
	**2.00**	2341±79°	547±5^{mnop}	1156±63^{t}	6.37±0.05^{bc}	87.2±0.0^{ab}
	**1.50**	2683,5±30,4^{lmn}	298±17^{pq}	2372±36^{q}	6.37±0.05^{bc}	88.0±0.0^{a}
	**1.00**	2763±67^{klmn}	344±7^{pq}	2043±102^{r}	6.37±0.05^{bc}	78.7±12.1^{bc}
	**0.50**	2341±79°	547±5^{mnop}	1156±63^{t}	6.37±0.05^{bc}	87.2±0.0^{ab}
	**0.01**	1556±141^{p}	604±36^{lmno}	543±67^{u}	6.10±0.04^{fg}	78.7±12.1^{bc}
DWS	**2.50 (HT)**	2577±111^{mno}	1724±79^{de}	2815±78mo	5.40±0.00^{m}	76.3±11.3^{cd}
	**10.00**	2857±12^{jklm}	796±23^{jklm}	4376±45^{c}	5.50±0.04^{lm}	67.2±0.6^{de}
	**6.58**	2816±53^{klm}	725±13^{klmn}	4115±4^{de}	5.57±0.05^{l}	67.7±0.0^{de}
	**2.50 (S)**	2986±21^{hijkl}	734±15^{klm}	3942±45^{efg}	10.00±0.00^{a}	67.3±0.6^{de}
	**2.00**	3159±116^{fghij}	923±155^{ijk}	3785±70^{ghij}	10.00±0.00^{a}	67.7±0.0^{de}
	**1.50**	3498±118^{e}	1088±170^{hi}	3861±8^{fghi}	10.00±0.00^{a}	67.7±1.1^{e}
	**1.00**	4044±51^{cd}	1340±73^{fgh}	3987±168^{ef}	10.00±0.00^{a}	66.8±0.0^{de}
	**0.50**	3875±18^{d}	1010±107^{ij}	3654±16^{j}	10.00±0.00^{a}	68.1±0.6^{de}
	**0.01**	3257±21.2^{efghi}	417±114^{opq}	2922±25^{lm}	10.00±0.00^{a}	67.7±1.1^{de}
RMS	**2.50 (HT)**	2884±24^{jkl}	2131±18^{cb}	2602±35^{p}	5.77±0.05^{k}	67.8±0.6^{de}
	**10.00**	3331±56^{ef}	1503±26^{ef}	3966±45^{efg}	6.00±0.18^{ghi}	68.1±0.6^{de}
	**6.58**	3352±1^{ef}	1487±9^{ef}	3748±9^{hij}	6.00±0.10^{ghi}	67.3±0.6^{de}
	**2.50 (S)**	3264±202^{efghi}	1126±96^{ghi}	3677±115^{ij}	5.93±0.00^{hij}	67.7±0.0^{de}
	**2.00**	3275±58^{efgh}	819±39^{jkl}	3881±22^{fgh}	6.07±0.09^{fgh}	67.7±1.2^{de}
	**1.50**	3248±21^{efghi}	470±166^{opq}	3917±21^{fgh}	6.00±0.18^{ghi}	67.7±1.1^{de}
	**1.00**	3310±43^{efg}	222±21^{q}	3727±95^{hij}	6.00±0.00^{ghi}	67.3±0.6^{de}
	**0.50**	3323±32^{ef}	421±11^{opq}	2871±45^{lmo}	6.07±0.00^{fg}	66.9±0.0^{e}
	**0.01**	2712±9^{lmn}	612±6^{lmno}	1745±5^{s}	6.13±0.00^{fg}	67.7±0.0^{de}

Values are expressed as mean ± standard deviation. Different letters within a column indicate significant differences ($p < 0.05$). - no detectable results; CWF: control wheat flour; DWF: durum wheat flour; DWS: semolina durum wheat; RMS: Re-milled semolina; HT: high temperature treatment; S: standard treatment.
